# A Molecularly Complete Planar Bacterial Outer Membrane Platform

**DOI:** 10.1038/srep32715

**Published:** 2016-09-07

**Authors:** Chih-Yun Hsia, Linxiao Chen, Rohit R. Singh, Matthew P. DeLisa, Susan Daniel

**Affiliations:** 1 School of Chemical and Biomolecular Engineering, Cornell University, Ithaca, NY, USA

## Abstract

The bacterial outer membrane (OM) is a barrier containing membrane proteins and liposaccharides that fulfill crucial functions for Gram-negative bacteria. With the advent of drug-resistant bacteria, it is necessary to understand the functional role of this membrane and its constituents to enable novel drug designs. Here we report a simple method to form an OM-like supported bilayer (OM-SB), which incorporates native lipids and membrane proteins of gram-negative bacteria from outer membrane vesicles (OMVs). We characterize the formation of OM-SBs using quartz crystal microbalance with dissipation (QCM-D) and fluorescence microscopy. We show that the orientation of proteins in the OM-SB matches the native bacterial membrane, preserving the characteristic asymmetry of these membranes. As a demonstration of the utility of the OM-SB platform, we quantitatively measure antibiotic interactions between OM-SBs and polymyxin B, a cationic peptide used to treat Gram-negative infections. This data enriches understanding of the antibacterial mechanism of polymyxin B, including disruption kinetics and changes in membrane mechanical properties. Combining OM-SBs with microfluidics will enable higher throughput screening of antibiotics. With a broader view, we envision that a molecularly complete membrane-scaffold could be useful for cell-free applications employing engineered membrane proteins in bacterial membranes for myriad technological purposes.

The bacterial outer membrane (OM) is a distinctive feature of gram-negative bacteria^1^,^2^. It forms a semi-permeable barrier that protects the bacterium from environmental attack, allows essential nutrients to cross the membrane to sustain life, and plays a major role in the virulence of pathogenic bacteria. The ability of the OM to prevent the entry of many antibacterial agents, especially hydrophobic compounds, is closely related to its structure. The OM is highly asymmetric, with the outer leaflet mainly composed of lipopolysaccharides (LPS) and the inner leaflet consisting of phospholipids^3^,^4^. The tightly packed LPS molecules on the outer leaflet give the bacteria structural integrity and form a stable barrier that resists antibacterial penetration^1^,^5^. In addition to the lipid content of the OM, proteins embedded in this membrane also play important biological functions, such as omptins (*e.g.*, ompT) that facilitate signal transduction through the cell envelope^6^ and pore-forming toxins (*e.g.*, ClyA) in the OM that perforate and disrupt host cells^7^. The importance of the OM in these and other important biological processes urges the development of tools to (1) understand fundamental biological events occurring in the OM^8^, and (2) study the action of new antibacterial drugs against OM components^9^,^10^.

To circumvent the experimental complexity imposed by *in vivo* systems, we developed a versatile model system to study bacterial outer membrane molecules in a convenient planar geometry that is compatible with many bioanalytical techniques and high throughput microfluidic devices. Supported lipid bilayers (SLBs), are thin planar bilayers that self-assemble during lipid vesicle rupture near a hydrophilic surface^11^,^12^, and are one of the most commonly used model systems to study cell membranes^13^. First, SLBs closely resemble cell membranes with the ability to protect the hydrophobic portion of membrane species to preserve their structure and functionality. Second, SLBs are amenable to surface sensing techniques and imaging tools due to their planar geometry, enabling studies of fundamental biological interactions between lipid-lipid, lipid-protein^14^, and pathogen-host^12^,^15^,^16^,^17^.

Unfortunately, developing a SLB platform that closely mimics the bacterial OM environment is a challenging task for several reasons. First, it is difficult for negatively-charged LPS to form a planar bilayer sheet on a negatively-charged bare glass surface. Several strategies have been reported in literature to overcome this obstacle; such as depositing LPS monolayer on alkanethiol-linked gold surface^18^, or inducing LPS vesicle fusion on positively charged polymer polyethylenimine (PEI) supports^19^. However, these approaches employ surface modification to facilitate the formation of LPS monolayer/bilayer, which may result in artifacts due to the strong electrostatic/hydrophobic interactions between the modified interface and LPS layer. To avoid these complexities, Langmuir-Blodgett and Langmuir-Schaefer methods have been used instead. Recent work has shown that this approach can be executed to preserve the asymmetric lipid arrangement of the OM^20^. However, using this technique to incorporate OM proteins into the platform is not possible due to the processing of the materials at a hydrophobic/hydrophilic interface and the layer-by-layer approach used to rebuild the membrane from its lipid constituents. Another approach is to extract lipid materials from bacterial membranes and reconstitute them into vesicles and supported bilayers^21^,^22^,^23^. As a result, these supported bilayers contain a mixture of outer and inner membrane components with no native proteins incorporated. However, detergent reconstitution methods can be used to generate proteoliposomes, but they may induce denaturation of membrane proteins and loss of function. Detergent can be avoided with cell disruption methods and fractionation of resultant proteoliposomes with sucrose gradients to isolate bacterial inner membrane vesicles and form planar bilayers^24^. In the end, most of these reconstitution/disruption methods suffer from either a loss of asymmetry of the membrane or lack of protein content, which are both important features of the OM. Hence, we sought to develop a simple and detergent-free method to incorporate molecularly complete OMs (i.e., containing lipids, LPS, and proteins) into a supported bilayer platform that preserves native asymmetry.

Our approach for creating an OM-mimetic supported bilayer (OM-SB) involves bacterial outer membrane vesicles (OMVs) as the source of OM materials. OMVs are nano-scale vesicles naturally secreted from gram-negative bacteria^25^. OMVs form during cell growth as the OM blebs outward and pinches off resulting in ~20–250 nm spheres of OM containing membrane-bound proteins and soluble periplasmic components trapped in their lumens^25^,^26^. Hence, the composition of OMVs reflects components of the OM and periplasm, for example, soluble proteins, OM proteins, and LPS. OMVs have been linked to a number of important biological processes such as envelope stress^27^, virulence^28^,^29^,^30^ and removal of antibacterial compounds^31^,^32^,^33^,^34^.

In this study, we created an OM-mimic platform by inducing OMVs to rupture into planar supported bilayers on glass/SiO_2_ supports. Unlike pure liposomes, OMVs are difficult to convert into supported bilayers via spontaneous vesicle fusion due to their high LPS and OM protein content^35^. To induce OMVs to fuse, we adapted a method reported previously by our group to form proteinaceous bilayers from mammalian cell blebs^36^. The resulting OM-SBs were studied in detail, including bilayer property characterization, protein orientation, and an investigation of bilayer formation kinetics using several surface-sensitive techniques: total internal reflection fluorescence microscopy, fluorescence recovery after photobleaching (FRAP), and quartz crystal microbalance with dissipation (QCM-D). Furthermore, we demonstrate using QCM-D that OM-SBs facilitate the evaluation of antibacterial drugs by providing details on mechanical property changes of membranes upon peptide-OM interactions. Using the OM-SB, we probed the antibacterial mechanism of polymyxin B (PMB), a cyclic cationic peptide used for treating gram-negative bacterial infection and endotoxin. The action of PMB on OM-SB was monitored using QCM-D in real time, and the data was subsequently fit to a theoretical model to quantify the change in mass and viscoelastic properties of OM-SB upon interaction with PMB. Based on the results, we validated that PMB interacts with the OM-SB following a mechanism of action that has been reported in literature^37^, supporting that the OM-SB is a suitable mimic of the bacterial membrane surface. However, with QCM-D, additional information can be learned about the changes in bacterial membrane properties (e.g., thickness, viscosity, shear modulus) upon peptide interaction, which we illustrate and report here for PMB. These mechanical properties are important for understanding how the outer membrane is compromised as a first protective layer preceding bacterial inner membrane failure.

To our knowledge, this work outlines the only approach to construct a molecularly complete planar bacteria outer membrane that includes bacterial proteins, lipids, LPS, etc., and that also preserves the hallmark asymmetry of the structure. By combining OM-SB with the appropriate surface sensing techniques, we illustrate the potential of an OM-SB as a convenient platform to quantitatively measure antibiotic interactions with bacteria membrane surfaces, including disruption kinetics and changes to bacterial membrane properties. Combining OM-SB with microfluidic platforms will enable higher throughput screening of compounds beneficial for future antibiotic design. Beyond thus, such a complete membrane-scaffold could be useful for cell-free studies/applications using expressed membrane proteins in bacterial membranes for myriad technological purposes.

## Materials and Methods

### Materials

A detailed list of materials used in this study is provided in the Supplemental Information.

### Methods

Details on the preparation of lipid vesicles, OMV size and surface charge characterization, Polydimethylsiloxane (PDMS) well fabrication, fluorescent labeling of OMVs and liposomes, preparation of glass coverslips used as supports for supported bilayers, formation of SLBs from pure liposomes, fluorescence recovery after photobleaching (FRAP), and Proteinase K susceptibility assays for the determination of ClyA-GFP orientation in OM-SB are provided in the Supplemental Information. In the following sections, we provide only the critical details on OMV preparation and characterization by QCM-D.

### Preparation of bacterial outer membrane vesicles (OMVs)

OMVs were purified as described in previously published literature^38^. Briefly, the plasmid pClyA-GFP was transformed into the JC8031 hypervesiculating strain of *Escherichia coli* and grown in Luria-Bertani (LB) medium supplemented with chloramphenicol (25 μg/mL) at 37 °C^39^. Protein expression was induced at an OD_600_ ~ 0.5 with the addition of L-arabinose (0.2% w/v) and allowed to grow for an additional 16 hours. Cells were pelleted at 7500 × *g* for 20 min at 4 °C and the supernatant was collected and filtered through a 0.02 μm filter. OMVs were then isolated from the filtrate via ultracentrifugation at 141,000 × *g* for 3 hours at 4 °C then resuspended in fresh buffer composed of 5 mM phosphate buffered saline (PBS) with 150 mM NaCl at a pH of 7.4, and stored at −20 °C. OMV protein content was quantified via bicinchoninic acid assay (QuantiPro BCA Assay; Sigma, USA) using a BSA protein standard (Sigma, USA).

### Quartz crystal microbalance with dissipation monitoring (QCM-D)

QCM-D was used to quantify the kinetics of various supported lipid bilayers formation. This technique measures changes of resonance frequency (Δf) and energy dissipation (ΔD) of an oscillating piezoelectric quartz crystal, which is driven by an applied AC voltage. The shift of resonance frequency (Δf) reflects the change of adsorbed mass on the quartz crystal sensor. The fundamental frequency (5 MHz) together with the third (15 MHz), fifth (25 MHz), seventh (35 MHz), ninth (45 MHz), eleventh (55 MHz) and thirteenth (65 MHz) overtones were generated and recorded during measurements. Simultaneously, shifts of energy dissipation (ΔD) were measured, which characterize the viscoelastic properties of the adhered layer to the crystal surface. Please see Supplemental Information for a detailed description of QCM-D models, including Sauerbrey Model^40^, One-layer Voigt-Voinova model^41^ and Two-layer Voigt-Voinova model^41^.

### QCM-D experimental setups for supported bilayer formation

All experiments were measured on QCM-D crystals made of silicon dioxide (QSX303, Q-Sense, Sweden) using a Q-Sense E1 (Q-Sense, Sweden) instrument. Before measurements, crystals were cleaned with Milli-Q water and ethanol, and dried with nitrogen gas. Crystals were then cleaned in UV-Ozone Procleaner (Bioforce, USA) for 10 minutes to remove any organic contamination. Note that although the crystals chosen here are the most similar material to the glass slides used in the microscopic experiments, the bilayer formation kinetics observed using QCM-D and fluorescent microscopy may be slightly different due to the variance in the elemental composition of surfaces.

Measurements were taken under flow conditions. Solution was pumped into the chamber by Peristaltic pump (Ismatec Reglo Digital M2-2/12, Q-Sense, Switzerland). The experimental details are provided below for different bilayer formations:

### SLB formation on quartz sensors from pure liposomes

PBS buffer was pumped into the system at a flow rate of 100 μL/min for 5 minutes to collect the baseline of frequency and energy dissipation shifts (i.e. Δf = ΔD = 0) of the crystal itself. Afterward, 500 μL of pure liposome solutions were pumped into the flow chamber under 100 μL/min. Then, PBS buffer was sent through the chamber at 100 μL/min to wash the bilayer to achieve stabilized final frequency and dissipation shifts.

### OM-SB formation on quartz sensors

PBS buffer was pumped into the system at a flow rate of 100 μL/min for 5 min. Thereafter, 500 μL OMVs solutions were sent into the flow chamber under 100 μL/min. The solutions were circulated in the system until desired values of Δ*f* and ΔD were reached. Then the system was rinsed with PBS buffer for 10 minutes to wash out excess OMVs. 500 μL of PEG (5 k) 0.5% DOPC liposome solution was then pumped into the flow chamber under 100 μL/min until Δf and ΔD reached steady state. PBS buffer was then sent through the system to wash the bilayer to achieve stabilized final frequency and dissipation shifts.

After measurements, the system was rinsed with 0.1% sodium dodecyl sulfate (SDS, Sigma, USA) solution, Milli-Q water and ethanol to ensure the removal of any residual materials prior to the next experiment. Air was pumped through to dry the tubing and the flow chamber. The crystal was removed and cleaned by UV-Ozone for 20 minutes and sonicated for 2 hours at 40 °C.

### Peptide interaction with OM-SB on quartz sensor

To prepare peptide solutions, Polymyxin B sulfate salt (Sigma, USA) was dissolved in PBS buffer at the concentrations of 0.1 mg/ml. Following the formation of OM-SB, peptide solution at desired concentration was added in the system. The solution was flown until the signals stabilized (~1 hr), and replaced with a buffer rinse to achieve final stable values of frequency and dissipation (~30 min). The flow rate was set to 100 μL/min throughout all experiments.

To better understand the action of polymyxin B on OM-SBs, normalized changes of frequency and dissipation at various overtones (3^rd^, 5^th^, ..., 13^th^) upon addition of peptide solution were monitored and fit to a two-layer Voigt-Voinova viscoelastic model. Signals of fundamental resonance, i.e. F_1_ and D_1_, were discarded since they are rather unstable due to edge effects^42^.

## Results and Discussion

### Formation of OM-SB from OMVs

To induce OMVs to fuse to a glass surface, we modified a procedure reported previously by our group for the formation of proteinaceous bilayers from mammalian cell blebs^36^,^43^. This involved first adsorbing OMVs onto the glass followed by addition of PEG (polyethylene glycol)-liposomes to the system to catalyze OMV rupture. PEG-liposomes used here are composed of pure DOPC phospholipids mixed with a small fraction of PEGylated PE lipids containing PEG chains attached on their headgroups. There are several reasons for using PEG-attached liposomes. First, PEG cushion underneath the membrane can increase the limited aqueous space between the substrate and the lipid bilayer. The expansion of the water gap in between the bilayer and the support has been suggested to protect transmembrane proteins from denaturing during contact with the substrate and further preserves their functionality^44^, which promotes the potential use of OM-SBs for bacterial OM protein studies. Second, PEG is a hydrophilic polymer and thus closely mimics the hydrophilic structures of carbohydrates attached on lipid headgroups in the OM, such as sugar moieties linked to lipid A molecules.

**Figure 1:**
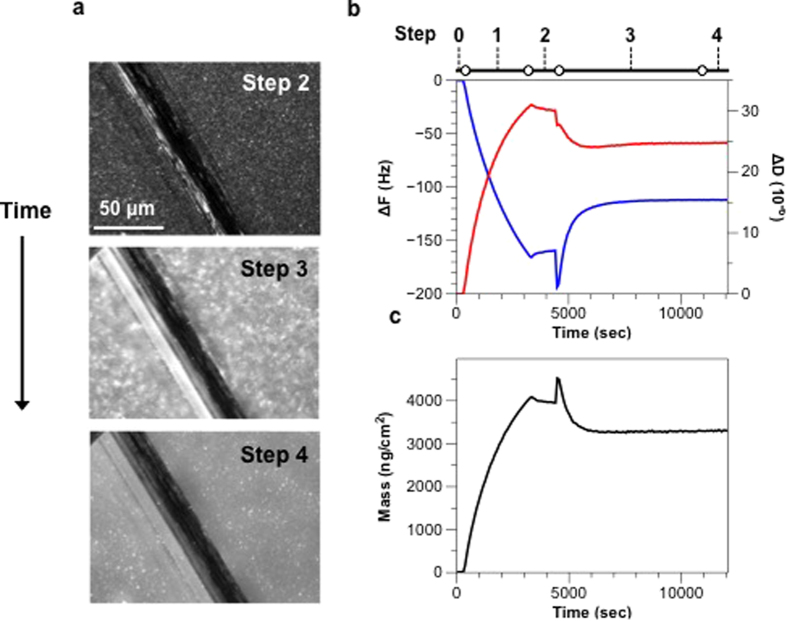
(**a**) The microscopy images showing the formation process of OM-SBs. OMVs were labeled with lipophilic fluorophore, R18, and PEG-liposomes were devoid of fluorescence. The dark lines in the images are scratches intentionally made to find the focal plane of bilayer. Intact OMVs labeled with R18 first adsorbed on the glass substrate (corresponding to step 2 in **b**). After the addition of PEG-liposomes, OMVs were induced to rupture (step 3), which resulted in the diffusion of R18 fluorophores from OMVs to newly formed bilayers (**b** step 3). The uniform distribution of the fluorescence indicated the contiguous nature of the OM-SB as well as the mobility of the R18 within it (step 4). The images were all taken under 40x magnification. A time-lapse movie of this rupture process can be found in the Supplemental Information. **(b)** Typical QCM-D curves showing the formation process of OM-SB. After initial PBS buffer baselines were achieved (0), OMVs were flowed into the chamber and adsorbed on the sensor (1). This step was followed by a PBS buffer rinse to remove excess OMVs not absorbed to the surface (2). PEG-liposomes were then sent into the system (3), which formed SLB patches and induced adsorbed OMV rupture. A final buffer rinse was made to remove any excess amount of vesicles from the system (4). **(c)** Frequency and dissipation signals were converted to adhered mass values, as shown in the lower plot. Changes in the mass on the surface along the formation process were determined using the one-layer Voigt-Voinova model (Supplemental Information).

To visualize the formation of OM-SB, we used fluorescence microscopy. OMVs were first labeled with R18, a lipophilic dye that intercalates into membranes. OMV solution was diluted to desired concentration in PBS. 50–100 μL labeled OMVs were incubated on a glass slide in a PDMS well for 15–20 minutes. OMVs adsorbed to the glass support observable as punctuate bright spots in the top image of Fig. 1a. Following adsorption, excess OMVs in the bulk solution were removed by rinsing the well with PBS buffer. OMVs adsorbed on the surface did not fuse and rupture into supported bilayers on their own. The immobility of lipids confirmed that bilayers were not formed at this stage (further confirmed by FRAP and QCM-D, discussed later).

To induce the OMVs to rupture, 50–100 μL of PEG-liposomes (2 mg/ml) were added to the PDMS well. The rapid formation of SLBs from PEG-liposomes in between the adsorbed OMVs catalyzed OMV rupture, most likely due to high edge energies of the SLB patches colliding with the adsorbed OMVs. The image series in Fig. 1a shows that the bright punctuate spots originally confined to the adsorbed OMVs had spread throughout the surface uniformly at this stage. Diffusion of R18 originating from the membranes of OMVs into the newly formed PEG-SLBs confirmed the rupture of OMVs. A time-lapse movie of this process can be found in the Supplemental Information.

### Acoustic property changes during planar bilayer formation

Quartz crystal microbalance with dissipation (QCM-D) is a technique that measures adsorbed mass to surfaces by tracking the change in resonance frequencies of the surface that is oscillated via piezoelectric excitation. Additionally, the dissipation feature measures the ability of the adsorbed material to dissipate acoustic energy, which can then be correlated to film stiffness. Figure 1b shows typical QCM-D frequency and dissipation responses and the corresponding mass curve of the OM-SBs formation process. First, OMV solution was sent to the chamber where OMVs gradually adsorbed on the QCM sensor, as indicated by the reduction in frequency and increase in dissipation. The amount of OMVs adsorbed depends on the length of time the solution is exposed to the sensor surface and the concentration of OMVs in the solution. A more detailed discussion of OMVs adsorption kinetics is provided in Supplemental Information. After the desired amount of adsorbed OMVs was achieved, PBS buffer was pumped in the chamber to rinse out unattached OMVs from the chamber. Buffer was then replaced by PEG-liposomes solution to induce OMV rupture. The frequency and dissipation changes that occur following the liposome solution support that OMVs ruptured upon the addition of PEG-liposomes, as was observed previously by monitoring R18 spread. After the frequency and dissipation shifts reached plateaus, PBS buffer rinsed out excess PEG- liposomes. Note that the final frequency was higher and dissipation was lower than the values before the addition of PEG-liposomes, indicating that not only PEG-liposomes form SLBs, but OMVs also rupture in the process.

### Diffusivity and mobility of OM-SBs

To further assess the mobility of OM-SBs, fluorescence recovery after photobleaching (FRAP) was used to measure two-dimensional diffusivity of the supported bilayers. Sample preparation and data analysis were performed as described in the Supplemental Information. Three types of samples were prepared: adsorbed OMVs (before rupture), formed OM-SB, and DOPC with 0.5% PEG(5K)-PE SLBs (PEG-SLB). All of the samples were labeled with R18 for performing FRAP experiments.

**Figure 2:**
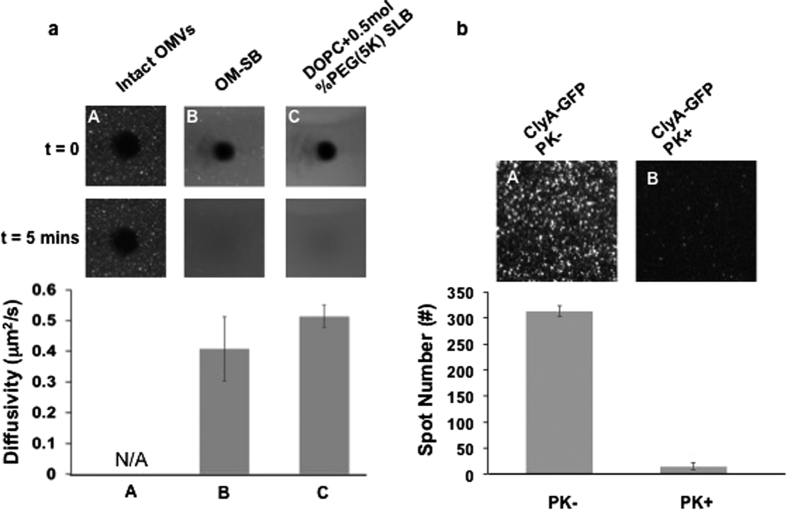
(**a**) Microscopic images of fluorescence recovery after photobleaching. (A) Intact OMVs labeled with R18. (B) OM-SBs labeled with R18. (C) DOPC with 0.5 mol% PEG(5K)-PE SLBs labeled with R18. The bar graph summarizes the diffusivities of these three types of supported bilayers. Note that due to the immobility of Sample A, no diffusivity could be determined. (**b**) Microscopic images of OM-SB expressing ClyA-GFP before (A) and after (B) Proteinase K treatment. The intensity of the punctate spots was abolished dramatically after the addition of proteinase K, which indicates that the majority of ClyA-GFP face up toward the bulk solution and that the orientation of the SB is the same as the OMV and bacterial cell. The bar graph below summarizes the densities of bright spots in the images. Multiple runs (n > 3) were performed to obtain bilayer diffusivities and particle numbers.

As shown in Fig. 2(a), Sample A contained only intact OMVs and the photobleached spot did not recover at all after five minutes. The result was expected since fluorophores can not diffuse across adsorbed, unruptured OMVs. Sample B and Sample C were OM-SB and PEG-SLB, respectively. The diffusivities of sample B and C were around 0.4 μm^2^/s and 0.5 μm^2^/s respectively. The mobile fractions were both near one, suggesting that lipid mixing between the PEG-SLB and OM-SB was complete.

### Orientation of OM-SBs

One of the motivations for developing an OM-SB is to use the resulting bilayers as mimics for the bacterial OM. Thus, it was imperative to determine the orientation of the resulting bilayer after rupture. This characterization is easily carried out using a green fluorescent protein (GFP)-based fusion protein expressed in the OMVs. Previously, we demonstrated that OMVs can be engineered to display recombinant proteins on their exterior by leveraging the vesicle-associated hemolysin ClyA as a carrier molecule^45^. Specifically, a genetic fusion between ClyA and GFP (ClyA-GFP) was expressed in hypervesiculating in *E. coli* cells and observed to localize in OMVs with GFP facing the external solution^38^.

To determine whether ClyA-GFP in OMVs became incorporated in OM-SB and oriented with extracellular side facing toward the bulk, proteinase K (PK) susceptibility experiments were performed on OM-SB derived from ClyA-GFP-containing OMVs in the following way. Upon exposure to PK, if the GFP proteins were oriented toward the bulk, they would be digested by PK and the fluorescence signal would be abolished. Otherwise the GFP would be protected beneath the lipid bilayer and resistant to PK treatment^45^. Figure 2(b) is the fluorescence microscopy of OM-SB derived from OMVs containing ClyA-GFP under 40x magnification, where the bright spots are the fluorescence signals from GFP. These images were analyzed using MATLAB (Mathworks) and ImageJ (NIH) for particle number counting. A home-based program was used to determine particle locations and numbers based on pixel clusters that meet an intensity cutoff^36^. We used a fixed particle size in the model, so any larger fluorescent spot, i.e. vesicle aggregation or clusters, will be counted as multiple particles depending on the surface area.

Before incubation PK with the OM-SB sample, there were 313 ± 10 particles in a 1,600 *μm*^2^ bilayer area (area shown in Fig. 2(b)). After the treatment of PK, the majority of the signals are eliminated, and the number of the punctate spots dropped to 15 ± 7 particles. The result suggests that greater than 95% of the GFP were oriented facing the bulk phase and proteolytically digested by PK in the solution. This protein orientation reflects that in the intact bacterial OM, as well as the orientation in the bacterial membrane itself. Note that the OM-SB in this experiment was created from pure DOPC liposomes without any PEG. The replacement was made as a precaution to remove the possibility that outward-facing PEG molecules adjacent to the bilayer might hamper PK ability to interact with the transmembrane proteins^46^ and bias the results.

### Kinetic analysis of adsorbed OMVs to OM-SB transition

With the ability to control the amount of OMVs adsorbed on the surface, we investigated the relationship between the adsorbed amount of OMVs and the quality of the resultant OM-SB; that is, the variation in the % of OMVs that rupture overall. A higher OMV rupture percentage indicates that fewer OMVs remain intact, which then results in higher OM-SB quality (fewer defects), and vice versa. To assess OM-SB quality, we first developed a method to estimate the surface coverage of adsorbed OMVs, the theoretical mass of supported bilayers from 100% OMVs, and the theoretical mass of OM-SBs. We then applied these analyses to calculate the OMV rupture percentage and assess the role of surface coverage on OM-SB quality.

### Estimation of the surface coverage of OMVs adsorbed on the sensor

To estimate the coverage of OMVs on the sensor, we formed a saturated monolayer of adsorbed, intact OMVs on the surface. The QCM-D curves in Figure S2 show that the mass at saturation of adsorbed OMVs (*M*_*satd*_) is approximately 12000 ng/cm^2^, which represents the maximum mass (jamming mass) for random sequential adsorption of OMVs on the surface. A detailed discussion of OMV adsorption kinetics is presented in the Supplemental Information. Thus, the surface coverage of unruptured OMVs (*θ*) can be estimated as a function of the mass of adsorbed OMVs (*M*_*Ad*_), the saturation mass of adsorbed OMVs (*M*_*satd*_). and the jamming limit of spheres on a 2D plane (~54%)^47^:


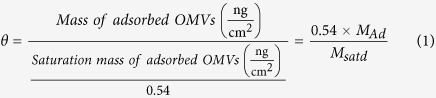


Note that the expression shown in eq. (1) is only valid under the assumption that the acoustic mass from QCM-D (*M*_*Ad*_, *M*_*layer*_) is proportional to the biomolecule mass, i.e. *M*_*Ad*_ ∝ Number of OMVs adsorbed. However, the amount of coupled water may change with number of OMVs adsorbed and hence the acoustic mass obtained from QCM-D may not truly reflect the amount of OMVs adsorbed on the surface. To correct the error caused by the assumption, we applied a theoretical model proposed by Bingen *et al.*^48^ along with a three-dimensional COMSOL Multiphysics model to quantify the variation of solvation at different OMV surface coverage. The simulated results can then be implemented to correct the surface coverage calculated using eq. (1) (Supplemental Information). All the calculations below were performed based on the corrected OMV surface coverage.

### Estimation of theoretical mass of supported bilayer made only from OMVs

In the next step, we estimate the theoretical mass of lipid bilayer composed of 100% of OMVs (OMV-SB). It is not feasible to experimentally measure the mass of a pure OMV-SB since OMVs do not readily self-assemble into supported bilayers on their own. Assumptions can be made to estimate the mass of OMV-SB as follows. The acoustic mass of OMV-SB is composed of the lipid mass, the protein mass, and the mass of solvent coupled to them. Kaufmann *et al.*^21^ reported that the mass of 100 wt% POPC SLB was 464 ng/cm^2^ and the mass of a SLB consisting of 90 wt% POPC and 10 wt% *E. coli* wild type LPS was 563 ng/cm^2^. Since bacterial outer membranes contain approximately 1:1 of phospholipids to LPS (ratio by weight), we assumed the mass of lipids and their coupled solvent was approximately 960 ng/cm^2^.

The other contribution in OMV-SB mass is from membrane proteins. OMVs contain approximately 9:1 of lipids and proteins by weight, based on the BCA essay and Bradford protein assay. Therefore we assumed that the mass of membrane proteins and their coupled solvents is one tenth of the lipid mass, 96 ng/cm^2^. Note that we may be slightly underestimating the mass of the solvent coupled with membrane proteins here since proteins may possess higher amount of solvent than lipids. We then combined both masses and the mass of OMV-SB, M_OMV-SB_, was determined to be 1075 ng/cm^2^.

### Estimation of theoretical mass of OM-SBs

We then applied the analyses above to estimate the theoretical mass of OM-SB, M_OM-SB,T_, representing the mass of a complete bilayer with all OMVs and PEG-liposomes ruptured. M_OM-SB,T_ is composed of the mass of OMV-SB (M_OMV-SB_) and PEG-SLB (M_PEG-SLB_ = 722 ± 5 ng/cm^2^) as a function of OMV surface coverage (*θ*) and area correlation coefficient κ:


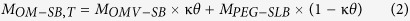



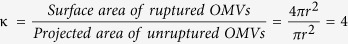


The coefficient κ illustrates the surface area changes of OM material on the support due to bilayer expansion upon vesicle rupture.

### Calculation of OMV rupture percentage

In the last step, we gathered all the information obtained above to calculate OMV rupture percentage. Ideally, if all adsorbed OMVs and PEG-liposomes ruptured, the experimental mass of OM-SB, M_OM-SB,EXP_, should be identical to the theoretical mass, M_OM-SB,T_. However, depending on the surface coverage of OMVs (*θ*), some amount of OMVs may not rupture, which then makes M_OM-SB,EXP_ different from M_OM-SB,T._

To determine the role of surface coverage of OMVs (*θ*) in shifting the OMV rupture percentage, we first analyzed the composition of the experimental mass of the OM-SB, M_OM-SB,EXP_ as follows:


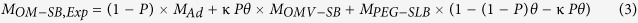



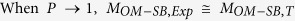


where *P* is OMV rupture percentage. The first term is given by the mass of non-ruptured OMVs. The second term is the mass of bilayer from the ruptured OMVs, and the last term indicates the mass of bilayer from PEG liposomes. Since all the variables are known except the rupture percentage (*P*), we can calculate its value from equation (3).

**Figure 3:**
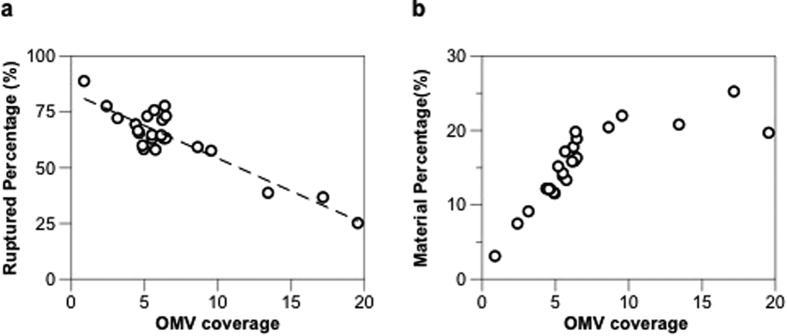
(**a**) The relationships of OMV surface coverage and the estimated OMV rupture percentage. The black dash line was a linear regression line with correlation factor, R^2^, to be 0.82. (**b**) The relationships of OMV surface coverage and the estimated OM material incorporated in OM-SB. Note that OMV coverage correlates to the footprint of intact OMV before rupturing. For instance, 9% OMV coverage with ~60% ruptured percentage corresponds to ~22% OM material in OM-SB.

Figure 3(a) reports the results of OMV rupture percentage (*P*) along with the corresponding OMV surface coverage (*θ*), showing the trend of lower rupture percentage for higher surface coverage. The inverse relation between the rupture percentage and OMV coverage is as expected. Lower coverage indicates higher PEG-SLB, which then increases the possibility for adsorbed OMVs to rupture upon contact with fluidic PEG-SLB. Moreover, it is less likely for PEGylated vesicles to get in and land on the spaces in between the intact OMVs at higher surface coverage. We concluded from the above analysis that fewer OMVs adsorbed on the surface would result in better OM-SB quality, that is fewer defects. Thus there is always a trade-off between the amount of OMVs adsorbed to obtain decent OM-SB quality and being able to incorporate a significant amount of OMV-originated materials into that OM-SB.

Based on the study of the estimated OMV rupture percentage reported in Fig. 3a, we have further calculated the corresponding amount of final OM component incorporated in OM-SB, as shown in Fig. 3b. Our calculation suggests that at lower OMV surface coverage (<10%), the amount of OM material in OM-SB increases as more intact OMVs are introduced in the system. When OMV surface coverage exceeds ~10%, the amount of OM materials that can be incorporated in the platform roughly levels out to 22–25%. Although beyond the scope of this study, we believe it may be possible to increase the amount of OM materials incorporated in OM-SB using several physicochemical approaches. By optimizing temperature, pH (especially acidic condition)^49^, liposome compositions and liposome sizes, we can potentially improve the quality of OM-LB with more OMV catalyzed to rupture and fuse.

### Kinetics of OMV rupture catalyzed by the addition of PEG-liposomes

Following the analysis of bilayer properties, orientation, and quality, we further analyzed QCM-D curves to characterize OM-SB formation kinetics. As shown in Fig. 1c, the mass curve displays four stages: OMVs adsorption (1), PBS rinse (2), PEG liposomes incubation (3) and final buffer rinse (4). To study how OM-SB forms upon the addition of PEG liposomes, we focus on the third stage since it reveals detailed information about the planar bilayer formation process.

**Figure 4:**
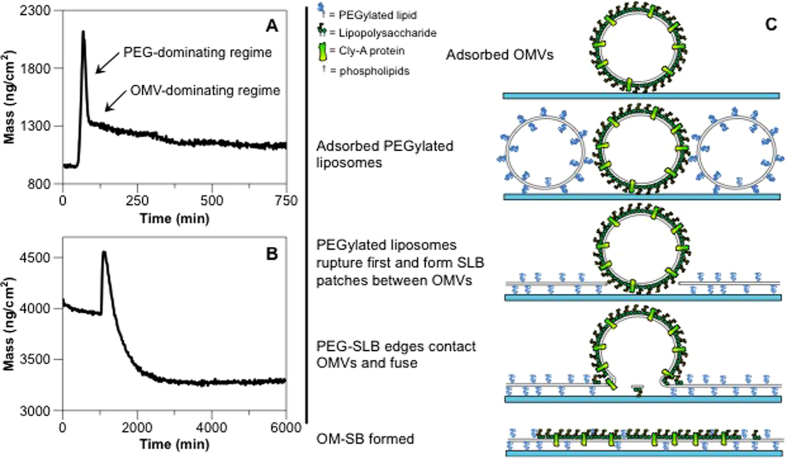
The mass curves of the formation process of OM-SBs with (**A**) lower OMV coverage (3.2%) and (**B**) higher OMV coverage (13.4%). (**A**) At lower OMV coverage, the rupturing process presents two-regime kinetics, the mass first dramatically decreases (PEG-dominating regime) and then transitions to a slower rate of decay (OMV-dominating regime). (**B**) At higher OMV coverage, only one-regime kinetic (OMV-dominating regime) is observed: the initial quick drop is no longer distinguishable. (**C**) A proposed scheme of OM-SB formation mechanism. OMVs first adsorbed on the substrate and remained in vesicle form. PEG-liposomes were then added to the system, which rapidly adhered to the surface and formed PEG-SLB in between OMVs. The newly formed PEG-SLB induces OMV rupturing and spread on the surface, which results in the formation of OM-SB. Note that the components of the OMV are not drawn to scale, but simplified to illustrate the mechanism of OM-SB formation.

Upon the addition of PEG vesicles, the mass dramatically increased due to the adsorption of PEG vesicles on the surface in between the OMVs. The critical coverage was soon reached and PEG vesicles began to rupture, as indicated by the decrease in mass. The decrease in mass was mainly due to the release of buffer from the internal lumens of both the PEG vesicles and the OMVs. From a series of experiments, we observed that the trend of the decrease in mass is highly dependent on the OMV surface coverage (*θ*). When OMV surface coverage is low (Fig. 4A), the negative mass shift presents two-regime kinetics, where the mass first dramatically decreases and continues to drop at a slower rate until it reaches steady state. As OMV surface coverage increases (Fig. 4B) only one-regime kinetics is observed with the loss of the first rapid decrease in mass.

One of the possible mechanisms to explain the observations is that the first regime, which is only present when OMV surface coverage is low, indicates that the dominating mechanism initially is the formation of bilayer from the PEG vesicles (PEG-dominating regime). The following slower negative mass shift suggests that the newly formed PEG bilayer then catalyzes OMVs to rupture, which is the rate-determining step in the second kinetic regime (OMV-dominating regime).

When working with low OMV surface coverage, there is a larger surface area for PEG vesicles to adsorb, rupture and form bilayer sheets, and this PEG bilayer formation process is less likely to be affected by the adsorbed OMVs. Therefore, it is not surprising that the PEG bilayer formation phase is distinguished from the OMV rupture phase in the curve. As OMV surface coverage increases, the surface area for PEG liposomes to form bilayer is limited, which makes pure PEG bilayer formation no longer significant enough to be observed alone. The higher amount of adsorbed OMVs force the immediate interaction of the small PEG SLB patches with unruptured OMVs and catalyze them to rupture. Throughout the entire process there are no distinguishable kinetic phases, but a single convolution of both types together, resulting in a general slowdown of the overall kinetics due to the slower kinetics of OMV rupture. The proposed OM-SB formation scheme described above is summarized and illustrated in Fig. 4C.

Based on the proposed formation mechanism, we built a kinetic model to describe the OMV-dominating regime to understand how OMVs were catalyzed to rupture via their contact with the edge of PEG bilayer:


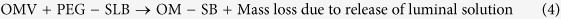


By hypothesizing equation (4) as a Poisson process, we fit the corresponding rate equation, a single exponential equation (5), to the normalized OMV-dominating regime, which represented the cumulative mass lost due to the release of encapsulated solution in OMVs:


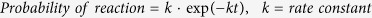


Poisson Process


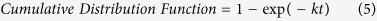


To define the location of the OMV-dominating regime, we followed the principles described next. We found qualitatively from a series of experiments that two-phases regimes occurred whenever surface coverage (*θ*) was lower than 10%, and we defined the OMV-dominating regime by finding location that gave the best least-squares fit of equation (5). For surface coverage (*θ*) higher than 10%, the mass curve starting from the maximum value till the plateau was seen as the OMV-dominating regime.

**Figure 5:**
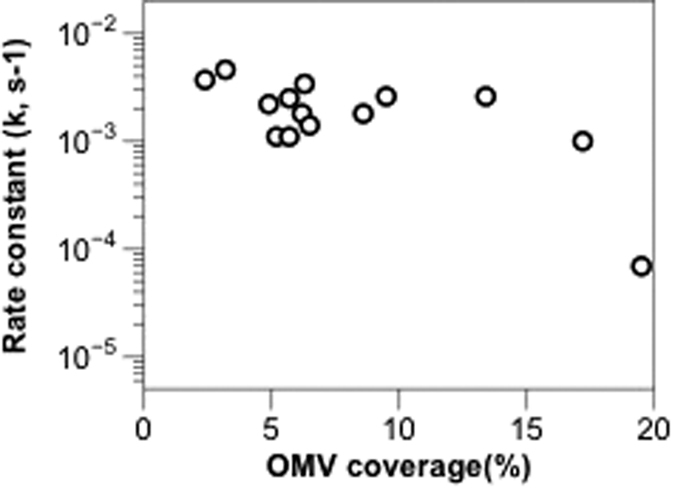
The rate constant k under different OMVs surface coverage.

Figure 5 shows the relationship between the rate constant, *k,* and OMV surface coverage, *θ*. When *θ *< 17%, the values of *k* were around 2 × 10^−3^ to 4 × 10^−3^ (1/s), showing no strong dependence on the surface coverage. However, once OMV surface coverage increased above 17%, *k* dropped significantly. The decrease in *k* at high surface coverage conditions may originate from the dense packing of OMVs. As *θ* increases, it is highly probable that OMVs are close enough together to limit the likelihood of OMVs contacting the PEG bilayers that have already formed. When OMVs no longer have access to freely associate with PEG bilayers, OMV rupture rate slows down and the rate constant *k* drops.

### Application of OM-SB: sensing anti-microbial peptide activity

The most valuable characteristic of OM-SB system is its native-like bacterial OM bilayer environment in the geometry of a planar sheet that couples well to analytical tools. Thus, OM-SBs open up many possibilities for investigating bacterial OM biological functions, especially its role in protecting gram-negative bacteria from its environment *i.e.*, antibacterial compounds. OM-SBs have great potential to test how bactericidal antibiotics disorganize/disrupt the highly ordered OM to kill gram-negative bacteria, which can be used as a basis for a new generation of antibiotic design.

In this study, we demonstrate how OM-SB is applied to probe the antimicrobial mechanism of polymyxin B (PMB), an amphipathic peptide composed of a cationic cyclic portion (a net charge of +5) and a hydrophobic fatty acyl tail. PMB is primarily used for treating gram-negative bacterial infection and endotoxin. The mechanism of action of PMB has been investigated since 1970s and because it has been studied extensively, PMB is a great candidate to test our OM-SB system against to ensure our system recapitulates known results and therefore is an effective, functional model of the OM surface. It is believed that PMB molecules specifically bind to lipid A molecules in a one to one ratio^50^ and the binding is generally described as following a two-step mechanism. The polar portions of PMB first interact with the anionic lipid A head groups through electrostatic attractions, which allows the aggregation of PMB on OM surfaces. These electrostatic interactions further promote the insertion of PMB’s fatty acyl chains due to their hydrophobic interaction with the lipid A tails^51^,^52^,^53^,^54^. The PMB-lipid A binding further alters membrane permeability by destabilizing the tight packing of adjacent lipid A fatty acyl chains to disrupt OM integrity.

To clearly elucidate the mechanism of PMB towards OMs, various techniques have been applied to determine the binding kinetics of PMB-lipid A complexes. For instance, Thomas *et al.*^18^,^55^,^56^ first reported the kinetics of each elementary step involved in the binding process using surface plasma resonance and stopped flow spectrofluorometry. However, to our knowledge, no kinetic evaluation of how the mechanical properties of the bacterial membrane change upon the binding of PMB has been reported. In this work, by monitoring the interaction of PMB and OM-SB using QCM-D, we are able to extract additional information on how bound mass and viscoelastic properties of OM vary during the interaction with PMB.

QCM-D has been used extensively to study antimicrobial peptide interaction with model membranes due to unique insights it provides into structural/conformational information of membrane-peptide complexes^42^,^57^,^58^. However, these studies were performed on simple artificial bilayers composed of phospholipids like POPC/DOPC, or biomimetic bilayers mimicking bacterial inner membrane (IM). Due to the difficulty of LPS vesicles to fuse into a supported bilayer^21^, to our knowledge, no QCM-D studies have been developed to study peptide-OM interaction. Since LPS is central to the mechanism of PMB action, investigating antimicrobial mechanism on OM-SB using QCM-D fills this gap in the literature.

To explore the interaction between PMB and OM-SB in a quantitative way, we fit the QCM-D responses using a two-layer Voinova viscoelastic model^41^. Most of the peptide-QCM studies use the Sauerbrey relation to directly correlate the frequency shifts to mass changes. However, as previously stated, OM-SB is a highly viscoelastic film (Δ*D* ≫ _10−6_) so that a viscoelastic model must be applied for an accurate estimation of mass and viscoelastic properties of the system. The one-layer model relies on the assumption that the entire system is a homogeneous viscoelastic film attached to the surface, so the fitted results of film mass, viscosity, and shear modulus represent a general picture of the mechanical properties of the whole layer. However, OM-SB is a heterogeneous film and we want to examine the detailed structural events occurring at different locations in the membrane. That is, for PMB binding/insertion kinetics, a two-layer model must be applied to distinguish mechanical property changes of the top layer (membrane surface at the interface with the bulk) from the bottom layer (the rest of the membrane). A detailed simulation scheme has been described in the Supplemental Information section.

Before investigating PMB activities, first we must define the location of the interface in between the layers. By fitting the frequencies and dissipations of all overtones at the initial time point to the model, we extract the information of thickness, viscosity, and shear modulus of both films. The simulated thicknesses of the top layer and the bottom layer are 3.6 ± 0.41 nm and 11.7 ± 3.40 nm, respectively. The model also yields a viscosity of 1.12 ± 0.17 cp and a shear modulus of (4.3 ± 1.4) × 10^5^ Pa for the top layer, and a viscosity of 1.34 ± 0.14 cp and a shear modulus of (4.6 ± 0.4) × 10^4^ Pa for the bottom layer.

**Figure 6:**
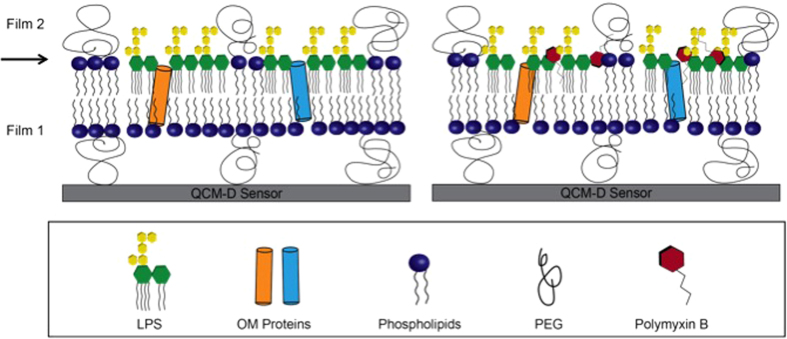
The illustration of (**a**) an OM-SB and (**b**) the complex of OM-SB-PMB. Based on the simulated result of two-layer Voigt-based model, an arrow was drawn to indicate the interface of film 1 and film 2. OM-SB contains both native OM lipids and a variety of OM proteins.

Based on the simulated results obtained from the two-layer model, the top layer extends from the headgroup of the upper leaflet to the PEG chain, and the rest of OM-SB then belongs to the bottom layer (as shown in Fig. 6a). Note that the PEG globular diameter is about 5 nm at this PEG density and the typical thickness of a lipid only bilayer is on order of 4 nm, so the model predictions fall within a reasonable range. The estimated thicknesses of both films also correspond well with the viscoelastic information obtained from the model. The viscoelastic ratio, tan(*δ*), of the top layer is about ten times smaller than the bottom layer, indicating the top film is particularly stiff compared to the rest of the system. The stiffness of the top portion is probably a result of dense packing of LPS molecules due to lateral interactions of sugar units and headgroups. This integrity further imposes steric constraints to the PEGlyated lipids and restricts the motion of the PEG chain above the bilayer as well. The rigidity of the LPS layer above the bilayer mechanically distinguishes the top glycosylated layer from the rest of the membrane bilayer.

**Figure 7:**
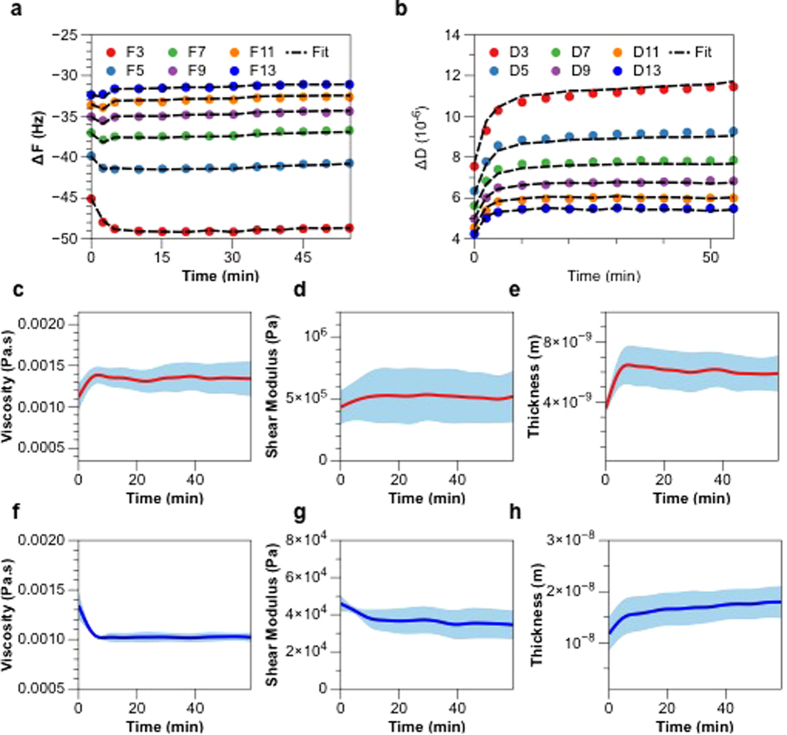
The experimental (**a**) frequency and (**b**) dissipation kinetics and the simulated results (black dot line) derived from two-layer Voinova model. As PMB is introduced into the system, the (**c**) viscosity and (**d**) shear modulus of the top layer (film 2, red) increase as PMB molecules bind with LPS and aggregate on the surface, and thus the mass of top layer ((**e**) thickness) also increases. Upon binding with LPS, PMB molecules further penetrate into the bilayer and disrupt its integrity, which then results in the decrease of bottom layer’s (film 1, blue) (**f**) viscosity and (**g**) shear modulus. (**h**) The mass of the bottom layer increases due to PMB binding and insertion. The simulation results were carried out using three individual experiments and the standard deviations are shown as the blue shaded regions.

An overall QCM-D response of OM-SB formation along with PMB addition is presented in Figure S5. The qualitative shifts of frequency and dissipation upon the addition of PMB solution are magnified in Fig. 7a,b. To rule out the possibility that non-specific artifacts (PMB to PEG-SLB) are present, negative control experiments were performed to confirm that no signal was detected with the addition of PMB to simple PEG-SLB (Supporting Information). We noticed that the frequency and dissipation kinetics are highly overtone-dependent. The lower-order overtones show decrease in frequency signals while the higher-order overtones exhibit a slight increase. The dissipation shifts grow for all overtones, and the increasing amount is in a descending order from low to high overtones. To further understand the physical meaning behind these responses, we fitted the frequency and dissipation data to the two-layer Voinova model and extracted the information of how viscosity, shear modulus, and thickness of both layers evolve along with the addition of PMB (Fig. 7c–h). These simulation results not only reveal important clues on the antimicrobial mechanism of PMB, but also provide a qualitative view of how PMB alters the OM-SB structure and mechanical properties.

First we pay our attention to the quick increase in thicknesses of both layers upon the addition of PMB. The increased thicknesses of both layers are probably a result of PMB-LPS complexes. Upon the introduction of PMB into the system, PMB molecules quickly bind to LPS (lipid A) and further penetrate into the bilayer with the hydrophobic tails inserted into LPS acyl chains. Since PMB-LPS complexes span across the layer-layer interface, the effective thicknesses grow for both the top and bottom films. The fitted changes of viscosity and shear modulus in both layers also support the proposed mechanism.

The aggregation of PMB on the bilayer surface and the tightly bound of PMB-LPS impose steric constrain to the top layer, resulting in the increase of shear modulus and viscosity of the top layer. Conversely, the shear modulus and viscosity of the bottom layer both dropped along with the OM-SB-PMB interaction. The insertion of PMB molecules disrupts the integrity of the bilayer and transforms the membrane to be more permeable to the bulk fluid. As a consequence, more solvent molecules are entrapped within the membrane, which lowers the shear modulus and viscosity of the bottom layer.

The above antimicrobial mechanism study successfully demonstrates that OM-SBs are a useful model that closely mimics OMs of bacteria, and it can be utilized as an *in vitro* platform to capture antibiotic mechanism of PMB. With the appropriate analysis, we are able to monitor how bilayer mechanical properties (thickness and viscoelasticity) evolve dynamically at different locations within the membrane. This detailed mechanistic information can be applied to propose antimicrobial mechanism of PMB, which turns out to correspond well with the existing studies (binding and insertion) in the literature for this compound. In addition, this platform serves as a unique tool to quantitatively report the mechanical property changes of the membrane itself induced by antimicrobial activity, which is not available by using other techniques.

As a final discussion point, we point out that at this stage we do not have a clear picture of whether the PEGylated lipids mingle evenly with the OMV components once the planar bilayer is formed, or if segregated domains form. Based on the microscopy/FRAP experiments is there is no evidence of R18 partitioning into domains, because the recovery curves do not show multiple populations and recovery is nearly complete. This suggests that there are no domains and good mixing between the PEGylated lipids and OMV lipids. However, since the R18 reporter is a single acyl chain with attached fluorophore, it may not accurately report how PEGylated lipids and LPS/proteins are organized/mixed. On the other hand, the polymyxin B experiments suggest that the interaction between the peptide and membrane surface are similar to what has been reported in the literature, so there does not seem to be a dilution effect due to the PEGylated lipids, suggesting that maybe there is no mixing after all. A final point to note is that by work conducted by the Kuhl Group on simple PEGylated SLBs using neutron and X-ray reflectivity suggests that the PEGylated lipids may form regions of cushioned SLB and uncushioned SLB^59^. We do not know if this structure would apply to our more complicated system with LPS, proteins, etc. However, blending these results, we can only conclude at this point that either: 1) the OM-SB is diluted with PEG-SLB and that concomitantly, the PEG-SLB has no effect on the native surface properties of the OM-SB, or 2) PEG liposome and OMV membrane are not well mixed and the resulting micro-domains cannot be observed by R18, either because it partitions equally well in both regions, or that at the observed “macroscale” of FRAP, the recovery looks uniform. A more thorough characterization of these bilayers will be attempted in the future, but the important point to emphasize here is that the platform recapitulates native peptide-OMV interactions and thus should be a useful platform for a variety of studies in the future.

## Summary and Conclusion

In this work, we developed an *in vitro* supported bilayer platform (OM-SB) directly from outer membrane vesicles (OMVs) of *E. coli* that closely mimics the outer membrane of these gram-negative microbes, including their native lipid and protein content, and preservation of the asymmetry of the membrane. Our approach, in contrast to reconstitution methods, overcomes the difficulty of incorporating OM lipids and proteins into a planar geometry while preserving native orientation and structure of membrane molecules.

The advantage of the planar OM-SB is its capability of being investigated by surface sensitive techniques. In this study, we combined both fluorescence microscopy and QCM-D to quantitatively characterize the OM-SB compositions and formation kinetics. We then demonstrated that the OM-SB is capable of recapitulating the mechanism of antibacterial agents using a well-study antimicrobial peptide, Polymyxin B, for an illustration. By elucidating the frequency and dissipation shifts measured using QCM-D with two-layer mechanical models, we provide new information as well about the changes in bacterial membrane mechanical properties during antibacterial action. We conclude that PMB binds to and aggregates on the OM-SB surface and destabilizes membrane integrity by further insertion throughout the bilayer. By extending similar studies to other antibiotic compounds using the OM-SB and surface analysis techniques like QCM-D, we believe this platform can offer many insights into understanding antibiotic kinetics for future drug design and screening host-pathogen interactions to facilitate bacterial vaccination development.

## Additional Information

**How to cite this article**: Hsia, C.-Y. *et al.* A Molecularly Complete Planar Bacterial Outer Membrane Platform. *Sci. Rep.* **6**, 32715; doi: 10.1038/srep32715 (2016).

## Supplementary Material

Supplementary Information
