# Supplementary material for: A Molecularly Complete Planar Bacterial Outer Membrane Platform
Source: Sci Rep. 2016 Sep 7;6:32715. doi: 10.1038/srep32715 (PMC5013322; doi:10.1038/srep32715)
Supplement: Supplementary Information [file srep32715-s1.pdf]

A Molecularly Complete Planar Bacterial Outer Membrane Platform

Chih-Yun Hsia, Linxiao Chen, Rohit R. Singh, Matthew P. DeLisa, and Susan Daniel*

School of Chemical and Biomolecular Engineering, Cornell University, Ithaca, NY.

SUPPORTING INFORMATION

1. Materials and methods used in: QCM-D modeling to detect adsorbed mass on the quartz sensor, Preparation of lipid vesicles, OMV size and surface charge characterization, Polydimethylsiloxane (PDMS) well fabrication, fluorescent labeling of OMVs and liposomes, preparation of glass coverslips used as supports for supported bilayers, fluorescence recovery after photobleaching experiments, and proteinase K orientation assay.
2. OMV and liposome characterization, size and charge. Figure S1.
3. OMV adsorption measurement using QCM-D. Figure S2.
4. OMV surface coverage discussion and adsorption simulation using COMSOL Multiphysics 5.2. Figure S3 - Figure S5.
5. QCM-D responses of all the overtones for OM-SB formation and the following polymyxin B interaction. Figure S6.
6. The QCM-D control experiment of adding polymyxin B to PEG-SLB. Figure S7.
7. A time-lapse movie file of the rupture of R188-labeled MVs with fusogenic vesicles.

S1. Materials and Methods

QCM-D modeling to detect adsorbed mass on the quartz sensor. QCM-D is a well-known technique to detect the adsorbed mass, Δm, on the crystal surface[1]. Several models have been developed to investigate mass and viscoelastic properties of the adsorbed film by fitting resonant frequency and dissipation signals:

- Sauerbrey Model

If the adsorbed film is rigid enough, which indicates that ΔD is smaller than 1x10^{-6} Hz, the Sauerbrey equation can be applied to obtain the adhered mass, Δm:

$$\Delta m = C_m \frac{\Delta f_z}{z} \quad (1)$$

Δm is the adsorbed mass on the crystal surface, C_m is a constant (-17.7 $\frac{ng}{cm^2 \cdot Hz}$ for crystal with f = 5 MHz), and Δf_z is the shift of frequency at z overtone (z = 1, 3, 5 , 7, 9, 11, 13). The Sauerbrey equation describes the linear relationship between the adsorbed mass and the change of resonant frequency for rigid adhered layers.

- One-layer Voigt-Voinova model

If the adhered layer is "soft" ($\Delta D > 1 \times 10^{-6}$Hz), the Sauerbrey equation is no longer valid. Instead, the Voigt-Voinova model is usually used to model the adsorbed mass along with several mechanical properties of the adlayer. The Voigt-Voinova model treats the adlayer and the rigid crystal as a coupling of a spring and a dashpot, with the assumptions of uniform adlayer thickness, uniform adlayer density, homogeneous viscoelastic properties and a no slip, Newtonian liquid in the bulk. The model includes four mechanical effects of the adlayer: thickness, density, shear elasticity and viscosity, and the equations are shown as following:

$$\Delta f \approx -\frac{1}{2\pi\rho_q h_q}\left[h_1\rho_1\omega - 2h_1\left(\frac{\eta_b}{\delta_b}\right)^2 \frac{\eta_j\omega^2}{\mu_1{}^2+\omega^2\eta_1{}^2}\right] \quad (2)$$

$$\Delta D \approx \frac{1}{4\pi f \rho_q h_q}\left[2h_1\left(\frac{\eta_b}{\delta_b}\right)^2 \frac{\mu_1\omega}{\mu_1{}^2+\omega^2\eta_1{}^2}\right] \quad (3)$$

$$\tan(\delta) = \frac{\omega\eta}{\mu} \quad (4)$$

Where ρ_q, ρ_f are the density of the crystal and film, h_q, h_1 are the thickness of the crystal and film, χ is the ratio of the storage modulus and the loss modulus, and δ is the penetration depth. $\tan(\delta)$ represents the ratio between the viscosity and shear modulus, which reflects the viscoelasticity of material. The smaller value of $\tan(\delta)$ indicates a more rigid material attached on the crystal, and vice versa.

We used the Voigt-Voinova model built in the commercial software, Q-tool, to obtain the adsorbed mass on the sensor since most of the data shown in this study contained the shift of dissipations (ΔD) much greater than $1 \times 10^{-6} Hz$. Two overtones of frequency shifts and dissipation changes, ($\Delta f_3, \Delta D_3, \Delta f_5, \Delta D_5$), were fitted to the Voigt-Voinova model to generate the information of thickness (h_1), shear elasticity and viscosity. The parameters used in the software were: film density (ρ_f): 1100 kg/m^3, fluid density: 1000 kg/m^3, fluid viscosity: 0.001 kg m^{-1}s^{-1}. The mass of the adsorbed film is the product of the film thickness and density:

$$\Delta m = h_1 \times \rho_f \ \left(\frac{ng}{cm^2}\right) \quad (5)$$

- Two-layer Voigt-Voinova model[2]

If the adhered layer is predominately heterogeneous, a two-layer Voigt-Voinova model can be applied to capture the detailed viscoelastic properties of different layered films. For two thin viscoelastic adhered layers in a bulk fluid, the changes in frequency and dissipation at various overtones are:

$$\Delta f \approx -\frac{1}{2\pi\rho_q h_q}\left\{\sum_{j=1,2}\left[h_j\rho_j\omega - 2h_j\left(\frac{\eta_b}{\delta_b}\right)^2 \frac{\eta_j\omega^2}{\mu_j{}^2+\omega^2\eta_j{}^2}\right]\right\} \quad (6)$$

$$\Delta D \approx \frac{1}{4\pi f \rho_q h_q}\left\{\sum_{j=1,2}\left[2h_j\left(\frac{\eta_b}{\delta_b}\right)^2 \frac{\mu_j\omega}{\mu_j{}^2+\omega^2\eta_j{}^2}\right]\right\} \quad (7)$$

By fitting frequency shifts and dissipation changes at various overtones ($\Delta f_3, \Delta D_3 \dots . \Delta f_{13}, \Delta D_{13}$) to two-layer Voigt-Voinova model, viscoelastic properties ($\eta_1, \eta_2, \mu_1, \mu_2$) and film thickness ($h_1, h_2$) of both top and bottom layers can be extracted. The fitting process was performed using an optimization tool of a commercial software package (MATLAB 8.3, The MathWorks Inc., Natick, MA, 2014a). Matlab's *fmincon* function was performed to find the minimum of the following constrained nonlinear multivariable function:

$$F(\eta_1, \eta_2, \mu_1, \mu_2, h_1, h_2) = \sum_{n=3,5\dots 13}\left(\Delta f_n^{exp} - \Delta f_n^{calculated}\right)^2 + \left(\Delta D_n^{exp} - \Delta D_n^{calculated}\right)^2 \quad (8)$$

Due to the nonlinear nature of the *F* function, various sets of fitted parameters were found, indicating the existence of multiple local minimums. To determine the most appropriate solution, we constrained the range of the viscoelastic parameters and film thickness to conform to physical constraints: $8.9 \times 10^{-4} Pa \cdot s \leq \eta \leq 1 \times 10^{-1} Pa \cdot s$, $10^4 Pa \leq \mu \leq 5 \times 10^6 Pa$, $1^{-9} m \leq \mu \leq 1^{-7} m$. Within the range specified, one specific set of fitted parameter was determined by the fitting tool to reach a global minimum. The density for both of the films was assumed to be 1100 kg/m^3.

Preparation of lipid vesicles. The lipids used in this study were DOPC (1,2-dioleoyl-*sn*-glycero-3-phosphocholine) and PEG (5K) - PE (1,2-dioleoyl-*sn*-glycero-3-phosphoethanolamine-N-[methoxy(polyethylene glycol)-5000]). Both lipids were purchased from Avanti Polar Lipids (Alabaster, AL). The composition of lipids used was: 0.5 mol% PEG(5K)-PE with 99.5 mol% DOPC. This composition was chosen so that the PEG would exist at (or below when mixed with OMVs) the mushroom-to-brush transition in the final bilayers to minimize interference with bacterial components and binding of peptides in later experiments[3]. The extension of the PEG chain at this composition is approximately 5 nm.[4] Lipids were dissolved and mixed in chloroform.

Chloroform was dried under high purity nitrogen gas and samples were subsequently placed in a vacuum desiccator overnight to drive off any remaining chloroform. Lipid films were rehydrated in PBS buffer to a final concentration of 2 mg/ml. Lipid solutions were extruded 15 times through a polycarbonate filter (Whatman Nucleopore) with 50 nm pore size and sonicated 20 min before use.

OMV size and surface charge characterization. The size and zeta potential of OMVs and pegylated liposomes in PBS buffer were measured by dynamic light scattering and electrophoresis (Malvern). The size distributions and zeta potential measurements are provided in the Supplementary Information.

Fluorescent labeling of OMVs and liposomes. In order to visualize the formation of bacteria-like SLBs and to measure the diffusivities of the subsequent bilayers, OMVs were first fluorescently labeled with Octadecyl Rhodamine (R18). R18 is a red-emitting fluorophore (Molecular Probes, Eugene, OR), which intercalates into membranes. 300 µL of OMV solution was incubated with 1 µL 0.18 mM R18 in a bath sonicator (Model # BD2500A-DTH; VWR) for 15-20 min on the lowest setting. The solutions were then centrifuged (Eppendorf, Centrifuge 5451C, Hauppauge, NY) through a G-25 spin column for 2 min to remove excess free R18.

Preparation of glass coverslips used as supports for supported bilayers. Glass coverslips were used as the substrate for supported lipid bilayers for fluorescence studies. 25 mm x 25 mm glass coverslips (No. 1.5, VWR) were cleaned in piranha solution for 10 min. Piranha solution consists of 45 ml 50% wt H_2O_2 (Sigma) and 105 ml H_2SO_4 (BDH chemicals). After cleaning, glass coverslips were rinsed with copious amounts of deionized water for 30 min. Deionized water was generated by an Ultrapure water system (Siemens Purelab). Clean glass coverslips were stored in deionized water and dried with nitrogen gas before each use.

Polydimethylsiloxane (PDMS) well fabrication. PDMS monomer and crosslinker were mixed in a ratio of 10:1. After being stirred and degased, the mixture was poured in a Petri dish and baked at 85°C overnight. The thin sheet of PDMS was then cut into small pieces such that they fit over the glass coverslips. Each piece had a hole punched at the center of diameter ~ 1 cm. The PDMS piece was attached on a clean glass coverslip to form a well to hold various solutions used here.

Formation of SLB from pure liposomes. SLBs self-assemble on clean glass by the vesicle fusion method[5]. Liposome solutions were added to a PDMS well and incubated for 10-15 min. PBS buffer was used to rinse the samples after incubation to remove excess lipid vesicles. In order to visualize the SLB formation and measure the mobility of lipids, liposomes (or OMVs) were labeled with R18 prior to bilayer formation, using the labeling procedure described above.

Fluorescence Recovery After Photobleaching (FRAP). To examine the diffusivity of lipids within the SLBs, FRAP experiments were performed using an inverted Zeiss Axiovert Observer. Z1 fluorescence microscope. The microscope was coupled with a α Plan-Apochromat 40x objective, a CCD camera (Hamamatsu ImageEM, model C9100-13, Bridgewater, NJ), a microscope light source (X-Cite 120, Lumen Dynamics Group Inc., Canada) and a Argon-Krypton tunable laser (CVI Melles Griot, model 643-AP-A01).

R18 was the fluorescence probe for all FRAP measurements conducted in this study. The SLB was photobleached with a ~10 μm diameter spot under 40x objective by the laser for 3 seconds. The fluorescence intensity of the bleached spot as it recovers with time was recorded for 15 minutes. To reduce artifacts resulting from background photobleaching, the fluorescence intensity of the spot was determined after background subtraction and normalization. The recovery data was then fit by following the method of Soumpasis[6]. The equation used to calculate the diffusivity is, $D = \frac{w^2}{4t_{1/2}}$, where w is the full width at half-maximum of the Gaussian profile of the focused laser

beam and $t_{1/2}$ is the characteristic diffusion time.

Proteinase K susceptibility assays for the determination of ClyA-GFP orientation in OM-LB

100 μg/mL of proteinase K (Ambion) for probing accessibility to the GFP domain of the ClyA-GFP protein) was added to ~120 μL samples of OM-SB (created from pure DOPC liposomes). Images of several regions of the surface were recorded before and after incubating proteinase K with OM-SB for 30 minutes. Particles of GFP were counted and the density of the spots were calculated, described in the *Results and Discussion* section.

S2. OMV size and surface charge characterization

The size and zeta potential of OMVs and pegylated liposomes in PBS buffer were measured by dynamic light scattering and electrophoresis. The size distributions of OMVs and DOPC with 0.5 mol% PEG (5k)-PE liposomes are plotted in **Figure S1a**, which shows that both vesicles are similar sizes with average hydrodynamic diameters around 70-100 nm. The zeta potential results (**Fig. S1b**) show that OMVs are more negatively charged than DOPC with 0.5 mol% PEG (5K)-PE liposomes in PBS. The negative charge of OMVs results from negatively-charged lipopolysaccharides, which makes up approximately 30 wt% of bacterial outer membrane, as well as protein content. We also used a ZetaSizerNano (Malvern) to determine concentration of the native OMV samples. Duplicate runs (n>3) were performed on each sample and three samples were measured. We found the correlation of OMV protein content (BCA assay) and particle number to be $1.21 \times 10^9 \pm 2.6 \times 10^8$ particles/ mg proteins. Figure S1c shows an example of concentration measurement using an OMV sample with 1.6 mg/mL protein concentration (BCA assay), with the average particle concentration to be $1.58 \times 10^9 \pm 1.27 \times 10^7$ particles /mL.

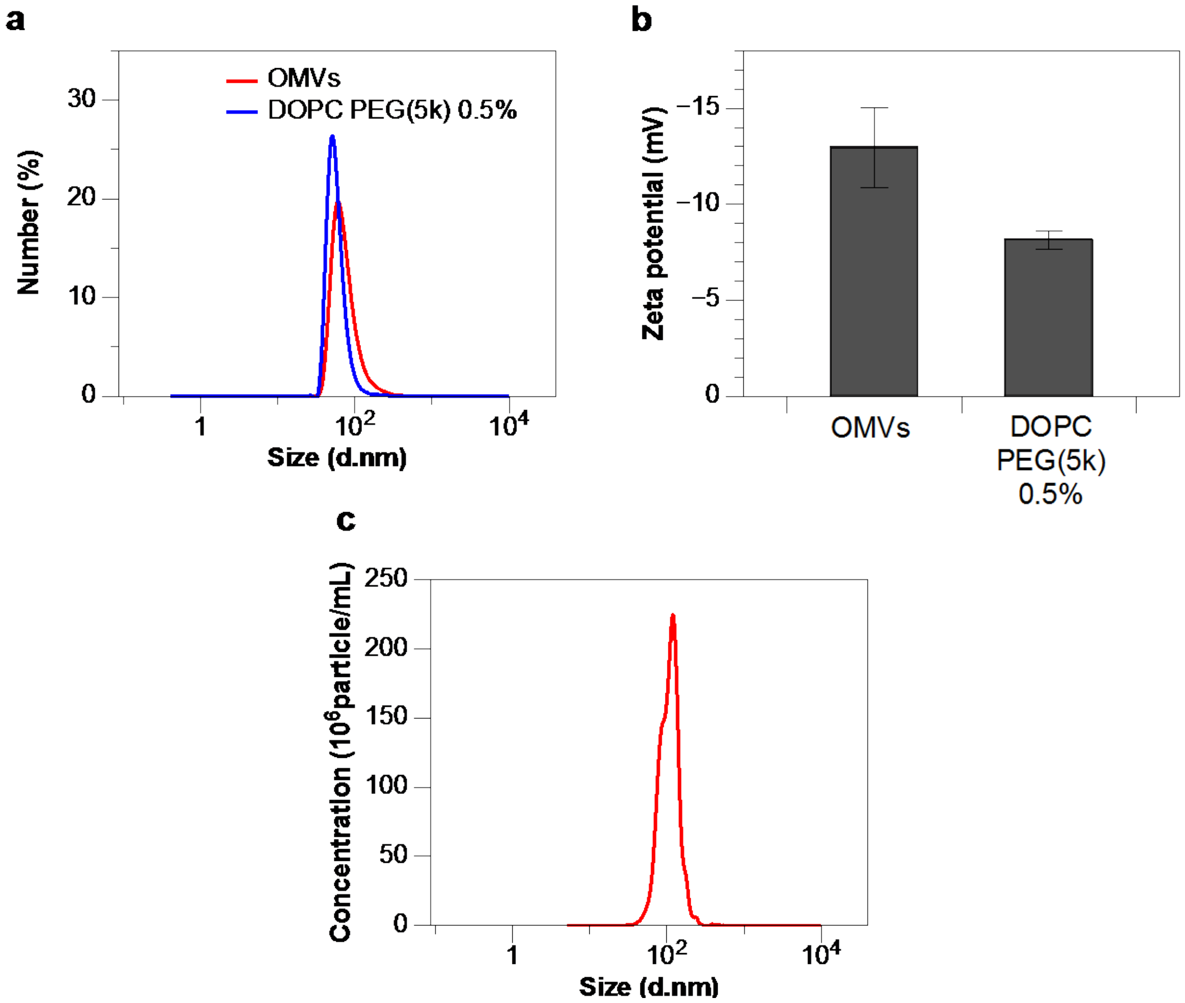

Figure S1. (a) Distribution of particle diameters and (b) zeta potential of OMVs and DOPC with 0.5 mol% PEG(5K)-PE liposomes in PBS buffer solution. Particle size distribution was determined by dynamic light scattering and zeta potential was obtained using electrophoresis. The buffer conditions were 5 mM phosphate buffered saline (PBS) with 150 mM NaCl at a pH of 7.4. (c) Particle concentration profiles for OMVs with 1.6 □ g/mL protein concentration.

S3. OMV adsorption measurement using QCM-D

To estimate the surface coverage of OMVs, we formed a saturated monolayer of intact OMVs on the sensor and monitored the adsorption process using QCM-D. The QCM-D measurement and the corresponding mass curve are shown in **Figure S2**.

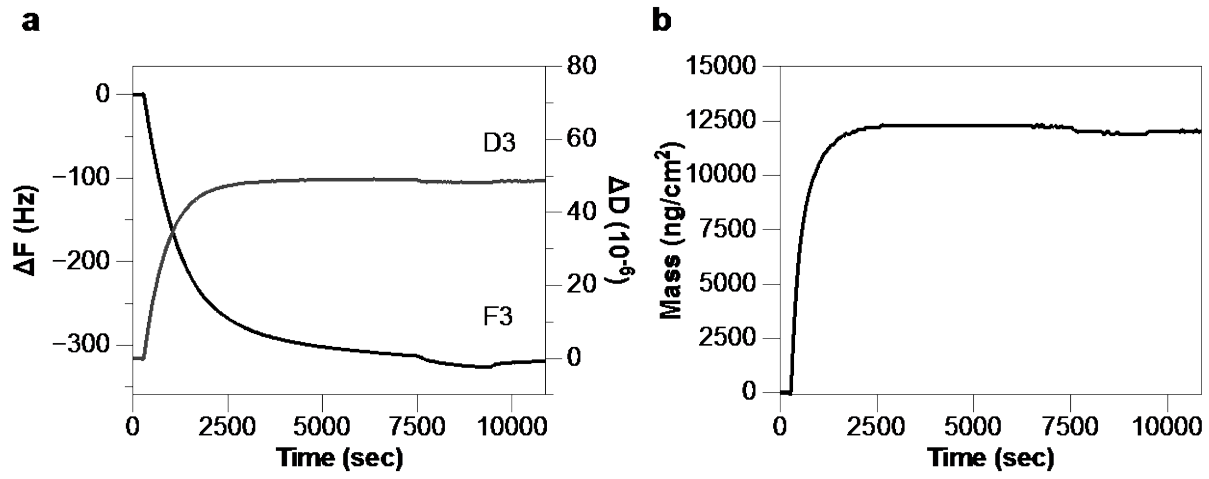

Figure S2. Typical result of a QCMD OMV adsorption experiment. While flowing, OMVs gradually adsorbed on the sensor, indicated by declined frequency and increased dissipation, and formed an unruptured OMV monolayer. The signals of frequency and dissipation were converted to mass curve (b) by fitting with one-film Voigt-Voinova model.

S4. OMV surface coverage discussion

The surface coverage calculation performed using eq. (1) in the main text was based on one simple assumption that the acoustic response obtained from QCM-D is proportional to the biomolecule mass. Note that acoustic response from QCM-D (M_{QCM}) is the combination of both dry biomolecule mass (M_d) and the coupled water mass (M_w):

$$M_{QCM} = M_d + M_w \tag{9}$$

By assuming M_{QCM} is proportional to M_d, we are stating that the hydration level, *H*, remains constant regardless of molecular level of OMV:

$$H = 1 - \frac{M_d}{M_{QCM}} = \ constant \tag{10}$$

$$\theta_H = \frac{0.54 \times M_{QCM}}{M_{QCM,sat}} \qquad \text{(eq. (1), main text)}$$

θ_H denotes the surface coverage calculated based on constant hydration (*H*) assumption. This assumption provides us a simple way to estimate OMV surface coverage, rupture percentage, and hence, the bacterial contents in OM-SB. However, previous literature has shown that this assumption is not valid under all circumstances. Cho et al. (2009)[7] have used complementary acoustic (QCM-D) and optical (SPR) techniques to monitor the hydration level of lipid vesicles adsorbed on a Au sensor. Their results suggest that the coupled water content per lipid vesicle varies at different adsorption stages. The hydration level stays high at low surface coverage and gradually decreases as more materials adsorbed on the surface. By assuming constant hydration level at all times, we will overestimate the biomolecular mass at low surface coverage, which will effectively distort the OMV rupture percentage calculation.

To further correct the artifacts caused by the assumption (eq (10)), we applied the theoretical model developed by Bingen et al (2008)[8] to describe the solvation of OMV at different surface coverage. Bingen and colleagues developed pyramid models to simulate the adsorption of vesicles on a QCM sensor. By assuming the space occupied by a vesicle with the solvent coupled to it to be a truncated pyramid, they were able to calculate the hydration level (*H*) as a function of surface coverage and parameters that describe the adsorbed vesicles:

$$H = \frac{v - M_w {\rho_p}^{-1}}{v + M_w({\rho_{H_2o}}^{-1} - {\rho_p}^{-1})} \quad (11)$$

$$v = func(\theta, r, z, l) \quad (12)$$

M_w is the molecular weight of the vesicle, ρ_{H_2o} and ρ_p are the density of buffer and particles, respectively. *v* is the average volume of the vesicle and its coated solvent, which is a function of surface coverage (θ) and parameters (*z*, *r*, *l*) describing the dimensions of the adsorbed vesicles. *z* is the height of the adsorbed vesicle, while *r* and *l* are the dimensions characterize the footprint of the truncated pyramid. To apply this theoretical model to describe the adsorption of OMVs, we

first need to obtain the following properties for the adsorbed OMVs: M_w, ρ_p, z, r, $and\ l$. We estimated M_w and ρ_p of the OMV to be 40,000 kDa and 1100 g/cm^3, assuming vesicle size in a range of with 70-100 μm and membrane thickness of 5 nm. We also estimated z, the height of the adsorbed vesicles, to be 110 μm based on the estimated thickness of OMV adsorbed monolayer from the Voigt viscoelastic model. However, we have very limited information on how to predict *r* and *l*, the other two dimensions describing the footprint of the adsorbed OMVs.

To overcome this barrier, we developed a three-dimensional COMSOL model to simulate OMV adsorption kinetics and further determine the parameters *r* and *l*. Note that while COMSOL model can successfully predict OMV surface coverage at different time courses, the model was developed under the assumption that vesicles were uniformly distributed. By not taking into account the random spatial distribution of adsorbed OMVs, we may miss significant OMV adsorption kinetics. This limitation motivates us to first fit the COMSOL simulation result in the "uniform adsorption" pyramid model (model 2 in Bingen et al) to determine parameters *r* and *l*. We then fit all the parameters to the modified pyramid model with random adsorbent distribution (model 3 in Bingen et al) to estimate the relationship of hydration level and surface coverage.

S4.1 OMVs adsorption on QCM-D sensor simulated using COMSOL

S4.1.1 Model development

I. Geometry

In the QCM-D system, the SiO_2 sensor (inner diameter 11 mm) was mounted in the liquid cell chamber (height 0.4 mm), and the chamber volume is around 40 μL. The geometry of the system is shown in **Figure S3**.

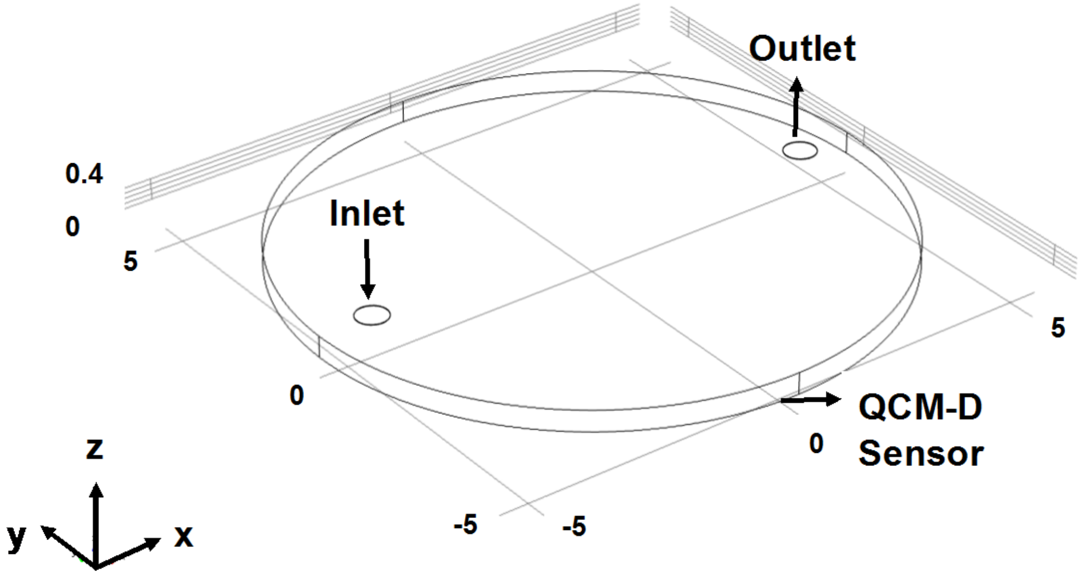

Figure S3: The geometry of the QCM-D flow chamber. The bottom surface is the adsorption surface site that generates frequency and dissipation changes. The OMVs solution were sent to the chamber through inlet and flowed out from outlet.

II. Physical Models

Three physical models were chosen in this study: Laminar flow (3D), transport of diluted species (3D), and surface reaction (2D). First, laminar flow physics was solved with stationary study, and the resulting 3D velocity profiles were input to transport of diluted species and surface reaction physics. The transport and surface reaction physics were solved in a time-dependent manner.

Physics 1: Laminar Fluid Flow

The flow in the chamber is laminar flow (Re ~ O(1)) and governed by Navier-Stokes equation:

$$\rho u \cdot \nabla \mathrm{u} = \nabla \cdot \left[-pI + \eta(\nabla \mathrm{u} + (\nabla \mathrm{u})^T) - \left(\frac{2\eta}{3}\right)(\nabla \cdot \mathrm{u})\mathrm{I}\right] \quad (13)$$

$$\nabla \cdot (\rho u) = 0 \quad (14)$$

u is solution velocity $\left(\frac{m}{s}\right)$. ρ (density $\left(\frac{kg}{m^3}\right)$) and η (viscosity $\left(\frac{kg}{m \cdot s}\right)$) are built-in properties of water at 24 ℃. P is the pressure (Pa).

The boundary conditions are: 1) Inlet: constant flow rate, 100 μL/min. 2) Outlet: P = 1 atm. 3) Walls: no flow, and the initial condition is: $u = 0$ (no flow at time zero).

The resulting continuous flow profiles are shown in **Figure S4**. The solution flowed in and exited out from the chamber at the velocity of 100 μL/min. The simulated results suggest that the flow rate experiences significant drop: 1) from the inlet/outlet to the center and 2) from the top to down along the z direction. This steady-state, unperturbed laminar flow profile was then used in the following studies to simulate OMVs adsorption on the sensor.

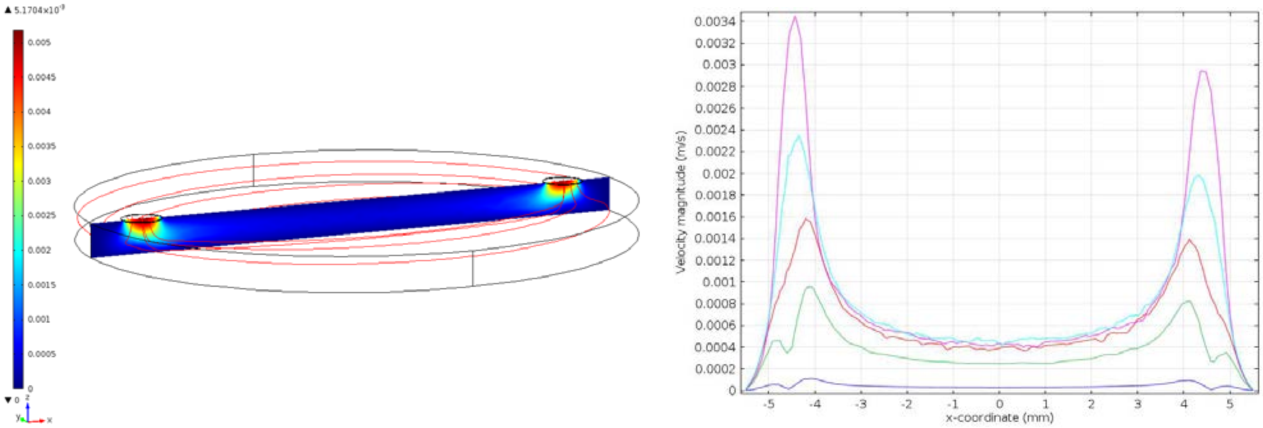

Figure S4. (Left) The magnitude of velocity field of the *zx* surface and the streamlines in the chamber. (Right) The magnitude of velocity field along (*x* = -5.5 mm, *y* = 0 mm) to (*x* = 5.5 mm, *y* = 0 mm) at different *z* slices, which corresponds to the surface showed in Figure 3. *z* ranges from 0.01 mm, 0.1 mm, 0.2 mm, 0.3 mm, and 0.4 mm.

Physics 2: Mass Transport in the stream

The governing equation of the transport of free OMVs (V) in the solution is described as below:

$$\frac{\partial C_V}{\partial t} + \nabla \cdot (-D_V \nabla C_V) + u \cdot \nabla C_V = 0 \quad (15)$$

where D_V is the diffusivity $(\frac{m^2}{s})$ and u is the velocity vector $(\frac{m}{s})$.

The boundary conditions are: 1) Inlet: $C_V = C_{V0}$, where C_{V0} was the solution concentration sent to the chamber. From t = 0 to t = 5 mins, the OMVs solution was fresh (C_{V0} = 0.01 mg/ml). For t > 5 mins, since the OMVs solution was recycled to the chamber, C_{V0} was the outlet

concentration, C_{out}. 2) Outlet: $n \cdot (-D_V \nabla C_V) = 0$. 3) Reactive sensor surface: $N_C = -R_{ad}$. 4) Other impenetrable surfaces: $n \cdot (-D_V \nabla C_V + C_V u) = 0$. The initial condition was $C_V = 0$, which means that the concentration of the bulk at the beginning of the process was set to zero.

Physics 3: Surface Reaction

The governing equation for OMVs adsorbed on the surface can be expressed as below, including OMV surface diffusion and the adsorption reaction:

$$\frac{\partial C_s}{\partial t} + \nabla \cdot (-D_s \nabla C_s) = R_{ad} \quad (16)$$

where C_s is the concentration of the adsorbed OMVs, D_s is the surface diffusivity and R_{ad} is OMV adsorption rate. If the surface diffusion was assumed to be zero, the governing equation for the surface concentration can be written as:

$$\frac{\partial C_S}{\partial t} = R_{ad} \quad (17)$$

Free OMVs (V) in the solution could adsorb on the surface site (*S*) on the QCM-D sensor irreversibly:

$$V + S \xrightarrow{K_{on}} VS \quad (18)$$

Where *VS* represents the OMVs adsorbed on the surface. The rate of adsorption can further be defined as:

$$R_{ad} = K_{on} C_V \Gamma_s (1 - \theta) \quad (19)$$

$C_V \left(\frac{mg}{m^3}\right)$ is the concentration of free OMVs in the solution on the surface, Γ_s is the total active binding site for OMVs (mg/m^2), and θ is the surface fraction of VS. Since both $K_{on}\ and\ \Gamma_s$ are unknowns in this study, we further combine these two parameters to one unknown constant A:

$$R_{ad} = A C_V (1 - \theta) \quad (20)$$

$$A = K_{on} \Gamma_s$$

By integrating eq. (16) and (20), the material balance for the adsorbed OMVs on the surface can be written as:

$$\frac{\partial c_S}{\partial t} = AC_V(1-\theta) \tag{21}$$

S4.1.2 COMSOL Simulation results and discussion

The constraint for the simulation model is that simulated surface coverage should saturate at the same time point as the experimental surface coverage, which is around t = 1600 sec under the given flow rate and solution concentration. By tuning the unknown parameter *A* in eq. 12, we are able to determine the surface coverage profile to reach saturation at the desired time. As shown in **Figure S5**, the experimental surface coverage data calculated based on the assumption (eq.(2)), θ_H, is higher than the simulated result, θ_{COMSOL}. This finding corresponds well with the proposed mechanism from the literature: the biomolecular mass will be overestimated at low surface coverage if hydration is considered to be constant. Fig. S4 also depicts the deviation of these two coverage profiles over the time course.

By expressing eq. 11 as eq. (22):

$$\theta_{model} = \theta_H \frac{1-H}{1-H_{sat}} \tag{22}$$

we can then fit the relationship of θ_H and θ_{COMSOL} to the "uniform adsorption" pyramid model (model 2 in Bingen et al) to determine the parameters *r* and *l*. The best fits are shown in **Figure S5a** and *r* and *l* are determined as 35 nm and 24.6 nm respectively. Finally, we fitted *r* and *l* to simulate the adsorption of OMVs by a random sequential adsorption (RSA) following a Monte Carlo algorithm[9]. The relationship between surface coverage $\theta_{model,\ RSA}$ and θ_H can then be computed (Fig. S5b) and used to correct the artifacts caused by the assumption (eq. (10)).

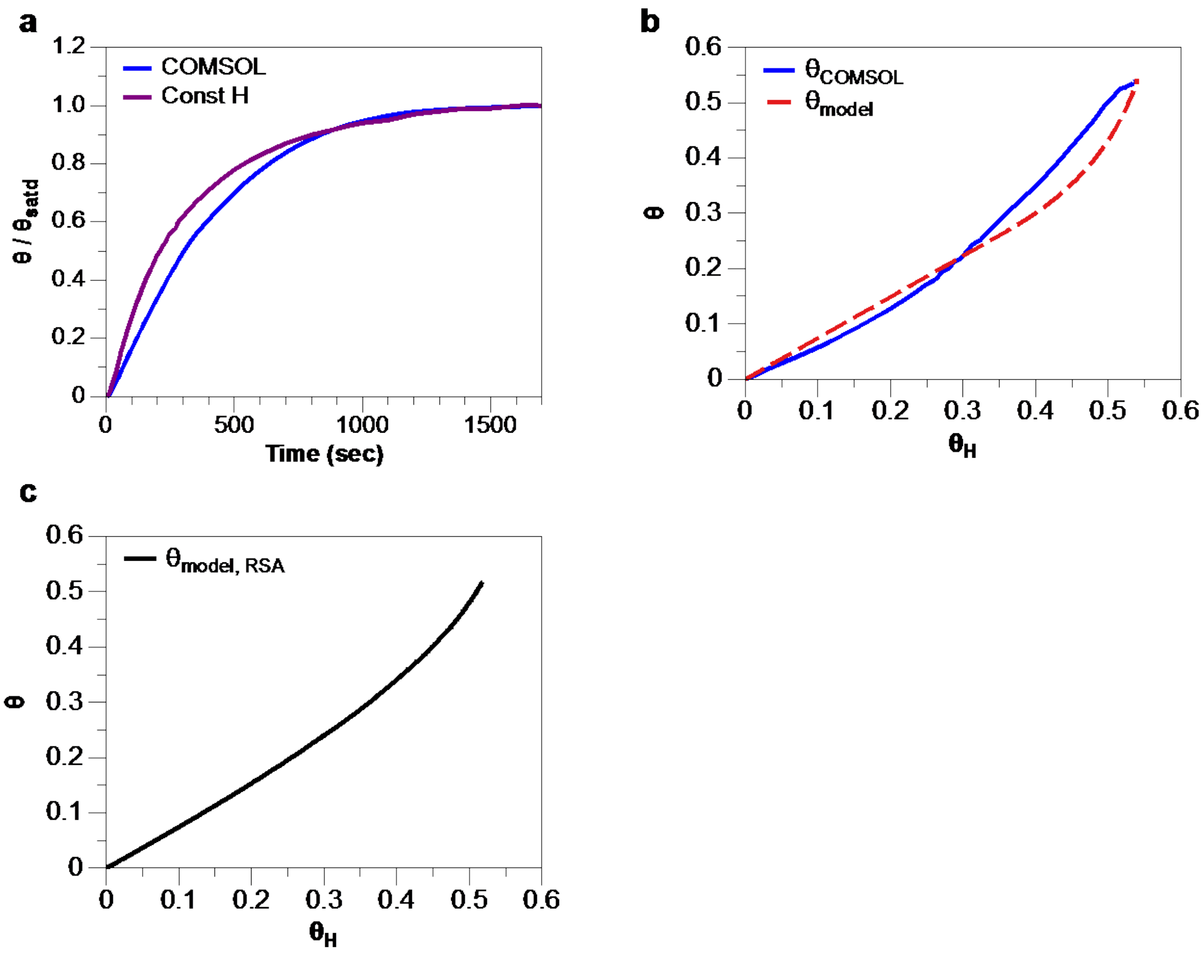

Figure S5. (a) The surface coverage from: (purple) the calculation using the experimental QCM-D data based on the assumption described in eq.10, and (blue) the simulation result using COMSOL model. (b) (blue) The relationship of θ_H versus θ_{COMSOL} and (red) the fitting results showing the relationship of θ_H and θ_{model}. (c) The final correlation curve used to correct the θ_H and $\theta_{model, RSA}$.

S5. QCM-D responses of all the overtones for OM-SB formation and the following polymyxin B interaction

We performed QCM-D experiments to monitor the interaction of polymyxin B towards OM-SB, ranging from the 3rd to 13th overtones. The QCM-D measurements including OM-SB formation and the following polymyxin B interaction are shown in Figure S6.

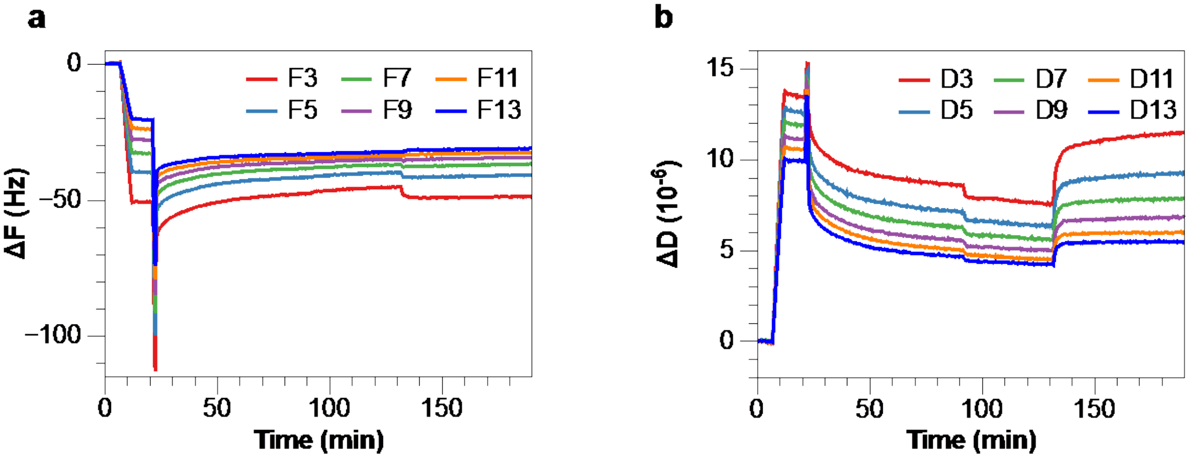

Figure S6. The 3rd to 13th overtones QCM-D responses for OM-SB formation and the following polymyxin B interaction. (Left) Normalized frequency response and (Right) the corresponding dissipation shifts.

S6. The QCM-D control experiment of adding polymyxin B to PEG-SLB

To exclude the possibility that non-specific artifact of polymyxin B to PEG-SLB are present, we performed QCM-D experiments to confirm that no signal was detected with the addition of PMB to PEG-SLB.

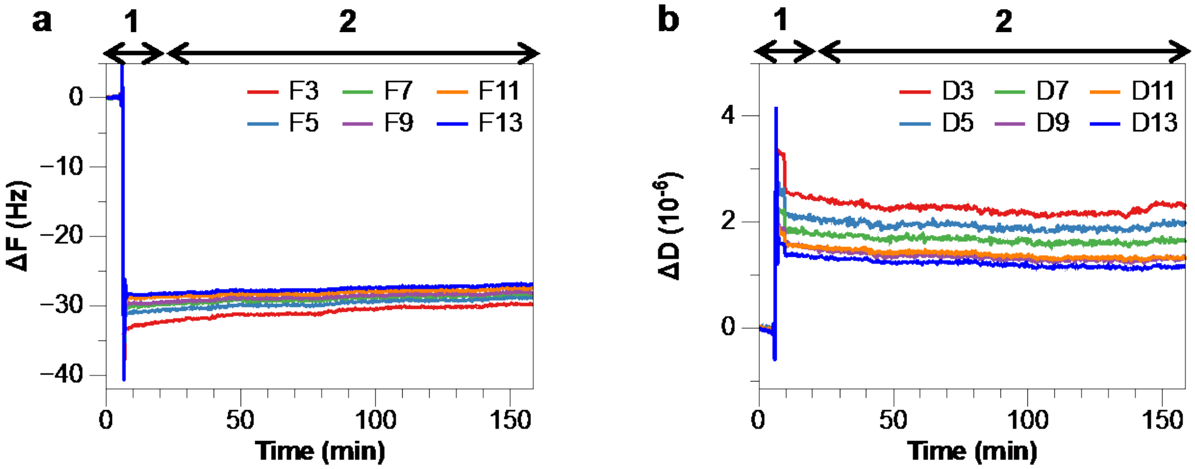

Figure S7. The QCM-D measurement showing PMB has no effect on PEG-SLB at the giving PMB concentrations. PEG-SLB was first formed (1) and PMB solution was then added into the system (2). The PMB concentrations range from: 0.001mg/ml, 0.01mg/ml, 0.05mg/ml, and 0.1mg/ml; each batch of solution was followed for 30 minutes. (a) Normalized frequency response and (b) the corresponding dissipation shifts.

S7. A time-lapse movie of the OMV rupture process to form an OM-SB.

Here OMVs are labeled with R18 and rupture to form a supported bilayer when fusogenic liposomes are added.

References

1. Cho, N.-J., Frank, C. W., Kasemo, B. & Höök, F. Quartz crystal microbalance with dissipation monitoring of supported lipid bilayers on various substrates. *Nat. Protoc.* **5,** 1096–106 (2010).

2. Voinova, M. V., Rodahl, M., Jonson, M. & Kasemo, B. Viscoelastic acoustic response of layered polymer films at fluid-solid interfaces: Continuum mechanics approach. **391,** 22 (1998).

3. Marsh, D., Bartucci, R. & Sportelli, L. Lipid membranes with grafted polymers: Physicochemical aspects. *Biochim. Biophys. Acta - Biomembr.* **1615,** 33–59 (2003).

4. Kaufmann, S., Papastavrou, G., Kumar, K., Textor, M. & Reimhult, E. A detailed investigation of the formation kinetics and layer structure of poly(ethylene glycol) tether supported lipid bilayers(1) Kaufmann, S.; Papastavrou, G.; Kumar, K.; Textor, M.; Reimhult, E. Soft Matter 2009, 5, 2804. *Soft Matter* **5,** 2804 (2009).

5. Richter, R., Bérat, R. & Brisson, A. Formation of solid-supported lipid bilayers: an integrated view. *Langmuir* **22,** 3497–3505 (2006).

6. Soumpasis, D. M. Theoretical analysis of fluorescence photobleaching recovery experiments. *Biophys. J.* **41,** 95–97 (1983).

7. Cho, N.-J. *et al.* Alpha-helical peptide-induced vesicle rupture revealing new insight into the vesicle fusion process as monitored in situ by quartz crystal microbalance-dissipation and reflectometry. *Anal. Chem.* **81,** 4752–61 (2009).

8. Bingen, P., Wang, G., Steinmetz, N. F., Rodahl, M. & Richter, R. P. Solvation effects in the QCM-D response to biomolecular adsorption - a phenomenological approach. *Anal. Chem.* **80,** 8880–8890 (2008).

9. Adamczyk, Z., Weroński, P. & Musiał, E. Colloid particle adsorption at random site (heterogeneous) surfaces. *J. Colloid Interface Sci.* **248,** 67–75 (2002).
